# Do Soil pH Levels Drive the Responses of Catalase Activity and Bacterial Communities to Microplastics? A Case Study in Mollisols

**DOI:** 10.3390/toxics13121005

**Published:** 2025-11-21

**Authors:** Yuan Yin, Xiangyu Wu, Qina Ren, Yuxin Guo, Zhonghui Yue, Xin Bai, Jia Xu, Pengwei Wang

**Affiliations:** College of Life Science and Technology, Harbin Normal University, Harbin 150025, China; 15046372485@163.com (Y.Y.); 18245031839@163.com (X.W.); 18845956404@163.com (Q.R.); guoyuxin97@163.com (Y.G.); 13845054490@163.com (J.X.); pengweiwang2001@163.com (P.W.)

**Keywords:** microplastics, mollisols, catalase, enzymatic reaction, bacterial community

## Abstract

Prolonged application and low recycling rates of agricultural plastic films have resulted in significant accumulation of microplastics (MPs) in soils, posing a threat to soil health. However, the impacts of MPs on microbial communities and enzyme activities in Mollisols remain poorly understood. To address the key question of whether soil pH drives the responses of catalase (CAT) activity and bacterial communities to MPs—a core focus of this Mollisol-based case study—we investigated the effects of different MP concentrations (1%, 5%, and 10%) on bacterial community structure and CAT activity across three Mollisol farmlands with distinct pH levels. CAT activity was stimulated at low MP concentrations but inhibited at high levels, whereas dynamic and thermodynamic parameters displayed irregular responses. Temperature sensitivity (*Q*_10_) of CAT remained stable, whereas *Q*_10_ of kinetic parameters varied among soils. Correlation analysis indicated that *E*_a_ and *Q*_10_ in acidic soil and *V*_max_/*K*_m_ in neutral soil and alkaline soil governed CAT activity. MPs altered *α*-diversity in acidic and neutral soils, changed *β*-diversity only in acidic soil, and promoted deterministic assembly processes. PICRUSt functional prediction suggested that functional gene shifts were most evident in acidic and neutral soils, with soil organic matter and *V*_max_/*K*_m_ as key drivers in acidic soils and CAT in neutral soils. In contrast, responses in alkaline soil were negligible. These findings highlight soil type-specific microbial responses to MPs and their ecological risks in agricultural soils.

## 1. Introduction

The application of agricultural plastic films has greatly improved crop productivity but has caused severe plastic pollution in farmland soils [1,2]. In China, the residual amount of agricultural plastic films in farmland currently accounts for approximately one-third to one-quarter of total usage, with an average residue of 60.0 kg hm^−2^ in the arable layer [3]. These films persist in farmland soils and degrade into fragments of various sizes under long-term cultivation, ultraviolet irradiation, and biodegradation, eventually forming smaller particles. When the particle size is less than 5 mm, they are classified as microplastics (MPs) [4]. MPs can migrate in farmland soils through abiotic processes (e.g., wind, erosion, surface runoff, leaching, and gravity) and biological mechanisms (e.g., activities of soil fauna, plants, and microorganisms) and gradually accumulate [5,6,7]. Owing to agricultural practices such as organic fertilizer application and plastic mulching, MPs accumulation is often more severe in agricultural soils [8,9]. Therefore, investigating the ecological impact of MPs in agricultural soils is crucial for assessing their environmental effects and ensuring sustainable agricultural practices [10].

Soil microorganisms and enzymes produced by microbes or plants are highly sensitive to foreign substances entering the soil and are widely recognized as important biological indices of soil quality [11,12,13]. As exogenous substances, MPs can alter the living environment of microorganisms by modifying soil physicochemical properties, thereby influencing both microorganisms and enzymes [14,15]. For example, Huang et al. reported that low-density polyethylene (LDPE) MPs increased the abundance of Bacteroidetes and Proteobacteria by altering soil porosity, water content, and dissolved organic matter (DOM) [16]. Liu et al. demonstrated that polyethylene (PE) MPs reduced the available carbon and phosphorus, resulting in resource limitations and strong environmental selection in soil bacterial communities [13]. De Souza Machado et al. suggested that MP-induced changes in soil bulk density and water-holding capacity could significantly affect microbial activity [17]. However, the extent of these impacts remains uncertain because of variations in shape, polymer type, and particle size. MPs also contain plasticizer components (e.g., dibutyl phthalate and bisphenol A) that are gradually released during degradation and can disrupt soil microbial communities [18,19]. In addition, MPs possess unique physical characteristics such as high surface area, rigidity, and hydrophobicity, which provide ecological niches for microorganisms and facilitate microbial colonization and biofilm formation, thereby altering soil microbial characteristics [16,20]. Several studies have examined the effects of MPs on soil enzyme activities. For instance, Huang et al. observed that the addition of LDPE fragments significantly increased urease (URE) and catalase (CAT) activities [16]. Conversely, polypropylene (PP) MPs inhibited dehydrogenase and sucrase activities but promoted URE activity [21]. Some nanoplastics can penetrate microbial membranes and enter cells directly, causing damage to microorganisms and altering the production and activity of soil-related enzymes owing to their extremely small size. Awet et al. reported that nanoscale polystyrene particles significantly reduced leucine aminopeptidase, alkaline phosphatase, β-glucosidase, and cell hydrolase activities in soil through controlled incubation experiments [22].

Among the diverse array of soil enzymes, CAT holds a unique position due to its central role in reactive oxygen species metabolism [23,24]. By catalyzing the decomposition of hydrogen peroxide (H_2_O_2_), it directly alleviates oxidative stress induced by pollutant exposure, making its activity a sensitive indicator of contaminant-induced toxicity [25,26,27]. Of particular importance, the aging of MPs and the leaching of additives may directly provoke oxidative stress, which establishes CAT as a critical bridge connecting MP pollution to microbial responses [18]. Thus, examining changes in CAT following the entry of MPs provides a valuable approach for assessing MPs-induced soil pollution under diverse environmental conditions. In recent years, soil enzymatic reaction characteristics, particularly kinetic parameters, have been recognized as effective indicators for monitoring soil pollution, offering important insights into the origin, catalytic properties, and behavior of soil enzymes [28,29]. Thermodynamic parameters of enzymatic reactions further reveal underlying mechanisms of enzyme catalysis and the characteristics of energy conversion during these processes. Previous studies on enzymatic reaction characteristics have largely focused on pollutants such as heavy metals and pesticides [30,31]. In contrast, the effects of MPs on soil enzyme kinetics and thermodynamics remain poorly understood.

Black soil is among the most fertile soils globally and the most suitable for agriculture, often referred to as the “giant panda of soils” [32]. As one of the three major black soil regions worldwide, the black soil area in Northeast China possesses unique fertility and significant advantages for agricultural development. It is also a vital grain-producing region and commercial grain base in China and plays a crucial role in ensuring national food security [33]. Owing to variations in surface water pH and differences in land development and utilization practices, soils with diverse pH levels have formed across the black soil region of Northeast China [34]. However, unsustainable agronomic practices have exacerbated the accumulation of MPs in this region, thereby creating widespread environmental concerns. Therefore, this study selected three representative northeastern farmland soils with different pH levels and conducted a 60-day incubation experiment. Three research questions were addressed: (1) Does CAT activity respond consistently to MPs across the three soils, and how do enzymatic reactions drive these changes? (2) Which aspects of soil bacterial composition, structure, and function are significantly altered by MPs? (3) Do the same factors drive the changes in three soil microbial communities?

## 2. Materials and Methods

### 2.1. Soil Samples and MPs

Three soils with different pH levels from the black soil region of Northeast China were selected as research objects. All three soils are typically cultivated Mollisols (USDA Soil Taxonomy). Acidic soil (S1, pH 5.41) was collected from the Yilan Green Vegetable Planting and Processing Cooperative in Heilongjiang Province, China (46°32′ N, 124°54′ E). This area is utilized for growing celery, tomatoes, cucumbers, and other vegetables under greenhouse cultivation. Neutral soil (S2, pH 6.64) was collected from the experimental park at Harbin Normal University, China (45°86′ N, 126°55′ E). This field is continuously cropped with soybeans under a soybean–corn rotation system. Alkaline soil (S3, pH 7.49) was collected from the silage crop experimental area in Daqing, Heilongjiang Province, China (46°27′ N, 129°64′ E). This field is cultivated with silage maize under continuous cropping. All three soil samples were collected manually with a shovel from the 0–20 cm soil layer. After natural air-drying, the soil was passed through a 2 mm sieve, and used for the chamber culture experiments.

Currently, LDPE is the most common mulch material in northeastern farmlands [35]. Therefore, it was selected as the test material in this study. LDPE was purchased from Zhonglian Plastic Chemical Co., Ltd. (Guangzhou, China). It exhibits an irregular spherical structure with a particle size range from 0.300 mm to 0.500 mm, a tensile strength of 300 kg cm^−2^, elongation at break of 500%, tensile modulus of 400 kg cm^−2^, density of 0.955 g cm^−3^, heat distortion temperature of 105 °C, and molding shrinkage rate of 0.37%.

### 2.2. Experimental Layout and Soil Incubation

MP concentrations were adjusted to 0% (*w*/*w*), 1% (*w*/*w*), 5% (*w*/*w*), and 10% (*w*/*w*), according to established methods. MP concentration in soil incubation experiments was very wide including from 0.05% to 20% (*w*/*w*) [36,37]. Scholars commonly conduct research using MP concentrations of 1% and 5%, in order to observe significant phenomena; some studies also used a MP concentration of 10% [38,39].

For each treatment, 200 g of soil was placed in a ceramic basin (top diameter 13.0 cm, height 12.0 cm), amended with MPs, thoroughly mixed, and equilibrated under natural conditions for 7 d. Each treatment was replicated three times, resulting in a total of 36 pots. Soil moisture was kept at 60% of the maximum field capacity and ultrapure water was added every three days during the incubation experiment. Finally, all pots were incubated under controlled conditions of 25 °C with a light regime simulating day–night cycles (16 h light/8 h dark) (PRX-600B, Hangzhou, China). The pots of all treatment groups were arranged using a completely randomized design, and a systematic rotation of all pot positions was carried out weekly to ensure the consistency of environmental conditions during the long-term cultivation. In a preliminary test, soil enzyme activities were measured on days 1, 3, 7, 15, 30, 60, 75, and 90. Enzyme activity peaked on day 60, after which it stabilized or declined by day 75 (Table S1). Based on these results, 60 d was selected as the incubation period to avoid loss of microbial activity. Following incubation, fresh soil samples were collected for physicochemical and enzymatic analyses (sieved through 1 mm), while the remaining soil was stored at −80 °C for microbial community analysis.

### 2.3. Soil Physicochemical Properties

Soil pH was measured using a pH meter at a soil-to-water ratio of 1:2.5. Soil organic matter (SOM) was quantified using the potassium dichromate oxidation external heating method. Total nitrogen (TN) was determined using the Kjeldahl method, and available nitrogen (AN) was measured using the alkaline hydrolysis diffusion method. Total phosphorus (TP) and available phosphorus (AP) were analyzed using the molybdenum–antimony anti-spectrophotometric method [40].

### 2.4. Soil CAT and Its Kinetic and Thermodynamic Analysis

CAT activity (EC 1.11.1.6) was determined at four temperatures (15 °C, 25 °C, 35 °C, and 45 °C) following the method of Guan [41].

For each assay, the soil samples with 60% water were incubated for 24 h in 100 mL flasks at the designated temperature. Hydrogen peroxide solutions with concentrations of 0.015, 0.030, 0.045, 0.060 and 0.075 mol L^−1^ were prepared. Water and H_2_O_2_ solution were added to the sample. After shaking for 20 min, the samples were titrated with potassium permanganate until a stable light pink color persisted for 30 s. A blank control without soil was included. The volume of potassium permanganate consumed was recorded, and CAT activity was expressed as the amount of permanganate consumed per gram of soil in 20 min.

The measured activity and substrate concentration were fitted to the Michaelis–Menten equation. Lineweaver–Burk plots (1/V versus 1/S) were then applied to calculate the kinetic parameters of CAT activity:

$$\frac{1}{V}\;=\;\frac{K_{m}}{V_{\max}}\text{×}\frac{1}{S}+\frac{1}{V_{\max}}$$

where V is the initial rate of CAT enzymatic reaction, *V*_max_ is the maximum rate of CAT enzymatic reaction, S is the hydrogen peroxide concentration, and *K*_m_ is the half-saturation constant.

CAT thermodynamic parameters were estimated by determining the potential enzyme activity (*k*) at different incubation temperatures for both MP-treated and untreated soils [42,43]. These parameters were then calculated using the following equations [44]:

$$k\;=\;A\;\times \;\exp(-\frac{E_{a}}{\mathrm{RT}})$$

$$Q_{10}=\frac{v_{T}+10}{v_{T}}$$

$$\Delta G=\mathrm{RTln}(\frac{\mathrm{RT}}{\mathrm{Nhk}})$$

where *k* is the potential enzyme activity (ml g^−1^) at a given temperature, A is the pre-exponential factor, *v*_T_ is the reaction rate at a given temperature, *v*_T_ + 10 is the reaction rate at a given temperature +10 °C, R is the gas constant (8.314 J K^−1^ mol^−1^), and T is the absolute temperature (Kelvin).

### 2.5. DNA Extraction, Amplification, and Sequencing

Soil samples (0.5 g) were extracted for total DNA extraction using the Soil DNA Isolation Kit (MoBio Laboratories, Carlsbad, CA, USA). The purity and quality of genomic DNA were examined on 0.8% agarose gel. An appropriate amount of DNA was then transferred into centrifuge tubes, quantified, and stored at −80 °C. Universal 16S rRNA primers 515F (GTGCCAGCMGCCGCGGTAA) and 909R (CCCCGYCAATTCMTTTRAGT) were used for amplification. The PCR cycling conditions were as follows: pre-denaturation at 94 °C for 5 min; 30 cycles of denaturation at 94 °C for 30 s, annealing at 55 °C for 30 s, and extension at 72 °C for 60 s; and a final extension at 72 °C for 7 min. Each sample was amplified in triplicate. PCR products from the same sample were pooled and separated on 2% agarose gel. Target bands were excised and purified using the AxyPrep DNA Gel Recovery Kit (Axygen Biosciences, Union City, CA, USA), eluted with Tris-HCl, and verified on 2% agarose gel. Quantification results were adopted to normalize PCR products to the required sequencing volume. Purified amplicons were sequenced in the paired-end mode on an Illumina platform (Analysis Pipeline Version 2.6, Allwegene Technology Co., Ltd., Beijing, China). Sequencing data were deposited in the NCBI Sequence Read Archive (SRA) under accession number PRJNA1276307.

### 2.6. Statistical Analysis

Measured enzyme activities, soil physicochemical data, and alpha diversity indices were subjected to one-way ANOVA (with prior confirmation that all datasets met the assumptions of normality and homoscedasticity via the Shapiro–Wilk test and Levene’s test, respectively), followed by least significant difference (LSD) tests (α = 0.05) to assess treatment effects (IBM SPSS Statistics 26, USA). Beta diversity was evaluated using Principal Coordinate Analysis (PCoA) based on the Bray–Curtis distance matrix.

The β-nearest taxon index (βNTI) was employed to infer microbial community assembly processes, and it was calculated using the iCAMP R package (v1.1.0). Briefly, the OTU table and phylogenetic tree were imported into R via the phyloseq package to construct a phyloseq object, followed by core taxa filtering (prevalence ≥ 25%, detection threshold = 0) using the core_members function from the microbiome package. The abundance data of the pruned phyloseq object was transposed to generate a community composition matrix, and the phylogenetic tree was midpoint-rooted with the midpoint_root function from the phytools package for cophenetic distance matrix calculation. βNTI values were finally computed using the qpen function from the iCAMP package (999 randomizations, 5 worker processes), where |βNTI| < 2 indicates stochastic dominance and |βNTI| ≥ 2 indicates deterministic processes [45,46]. Predictive functional potential of microorganisms was inferred from 16S rRNA gene sequences using PICRUSt2 (v2.5.2). The abundance tables of OTUs generated using QIIME2 were compared with KEGG (Kyoto Encyclopedia of Genes and Genomes) database to yield functionally predicted abundance [47]. The functional predicted abundance map was drawn and the significant differences in bacterial functions were analyzed and mapped in accordance with the functional predicted abundance of the level-1 and level-2 metabolic pathways of KEGG [48]. The Mantel test was used to examine correlations between environmental factors and bacterial community composition and diversity. All analyses were conducted in R (v4.1.2) using the “vegan” package.

## 3. Results

### 3.1. Soil Physicochemical Properties

The physicochemical characteristics of the three MP-treated soils are presented in Table 1. Following MP addition, soil pH increased in S1, decreased in S2, and remained unchanged in S3 (*p* < 0.05). Regarding soil carbon, MPs significantly reduced SOM in S1 and S2 (*p* < 0.05) but showed no effect in S3. For soil nitrogen, MPs did not alter TN in S1 and S2 but decreased TN in S3, whereas AN was reduced in S1 and S2 (except at 1% MPs), and only 1% MPs increased AN in S3 (*p* < 0.05). In terms of phosphorus, MPs decreased TP in all soils and AP in S2 and S3 (except at 1% MPs), whereas AP increased in S1 (*p* < 0.05).

### 3.2. Soil CAT Activity

**Figure 1:**
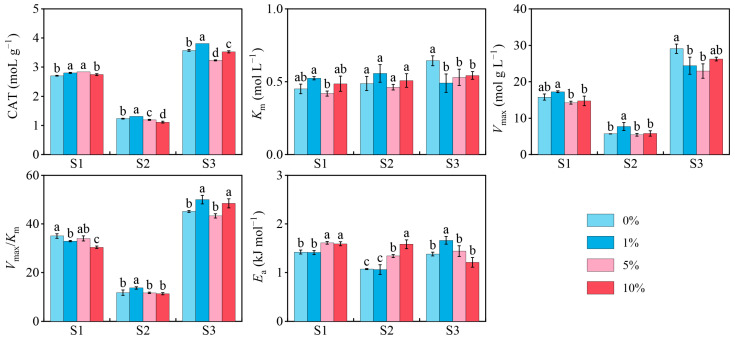
Catalase (CAT) activity, *K*_m_, *V*_max_, *V*_max_/*K*_m_, and *E*_a_ of CAT in three soils after microplastics (MPs) addition. Only the values of each index at the optimum temperature are shown. Data are presented as mean ± standard deviation of three independent replicate experiments. Lowercase letters indicate significant differences among MPs treatment groups within the same soil (*p* < 0.05).

Soil type, MP concentration, and their interactions significantly influenced CAT activity (Table 2, *p* < 0.05). At the optimum temperature (25 °C), 1% MPs increased CAT activity in all three soils, whereas 5% MPs enhanced CAT activity only in S1. In contrast, 10% MPs demonstrated no effect in S1 but reduced CAT activity in S2 and S3 (*p* < 0.05, Figure 1).

### 3.3. Soil CAT Kinetics and Thermodynamics

The kinetic and thermodynamic parameters of CAT under different MP treatments are presented in Figure 1. Significant effects were observed for soil type, MP concentration, and their interactions (Table 2, *p* < 0.05). The goodness-of-fit for all kinetic models was evaluated using the coefficient of determination (*R*^2^), with all resulting *R*^2^ values exceeding 0.86, thereby confirming the strong descriptive capability of the Michaelis–Menten model for the experimental data (Figure S1).

At the optimal temperature (25 °C), MPs exerted differential effects on the kinetic and thermodynamic parameters of the three soils (Figure 1). In S1, all MP treatments had no influence on *K*_m_ and *V*_max_ but significantly reduced *V*_max_/*K*_m_ (*p* < 0.05, Figure 1). Additionally, 5% and 10% MPs increased *E*_a_. In S2, *K*_m_ remained unaffected, whereas 1% MPs enhanced *V*_max_ and *V*_max_/*K*_m_, and 5% and 10% MPs simultaneously increased *E*_a_ (*p* < 0.05, Figure 1). In S3, MPs decreased *K*_m_ and *V*_max_, whereas *V*_max_/*K*_m_ increased under 1% and 10% MPs. Notably, 1% MPs increased *E*_a_ (Figure 1).

Regarding temperature sensitivity, MPs had no effect on *Q*_10_-CAT but significantly decreased *Q*_10_-*V*_max_/*K*_m_ in all soils (Table 3, *p* < 0.05). In S1, 5% and 10% MPs decreased *Q*_10_-*K*_m_ and *Q*_10_-*V*_max_, whereas these parameters increased in S2. In S3, only 5% MPs increased *Q*_10_-*K*_m_ and *Q*_10_-*V*_max_ (*p* < 0.05).

### 3.4. Soil Bacteria Diversity, Composition, and Assembly

Table 4 summarizes the changes in alpha diversity indices of the three soils under MP treatments. Both Chao1 and Shannon indices significantly increased in S1 but decreased in S2 across all MP treatments (*p* < 0.05). In contrast, no significant variation was observed in S3.

**Figure 2:**
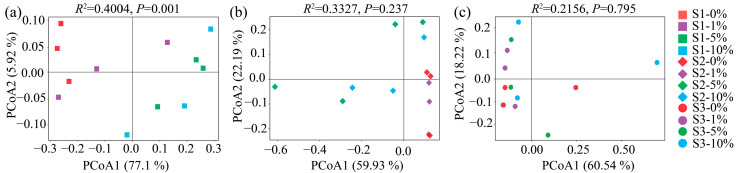
PCoA plots reflecting differences in bacterial community structure (**a**–**c**). Panels correspond to soils S1 (**a**), S2 (**b**), and S3 (**c**).

PCoA based on the Bray–Curtis distance was adopted to assess the influence of MPs on bacterial community composition at the OTU level (Figure 2a–c). MP concentration increase caused a clear separation of the bacterial community in S1 (Adonis: *R*^2^ = 0.4004, *p* = 0.001) (Figure 2a), whereas no significant separation occurred in S2 (Adonis: *R*^2^ = 0.3327, *p* = 0.237) or S3 (Adonis: *R*^2^ = 0.2156, *p* = 0.795) (Figure 2b,c).

**Figure 3:**
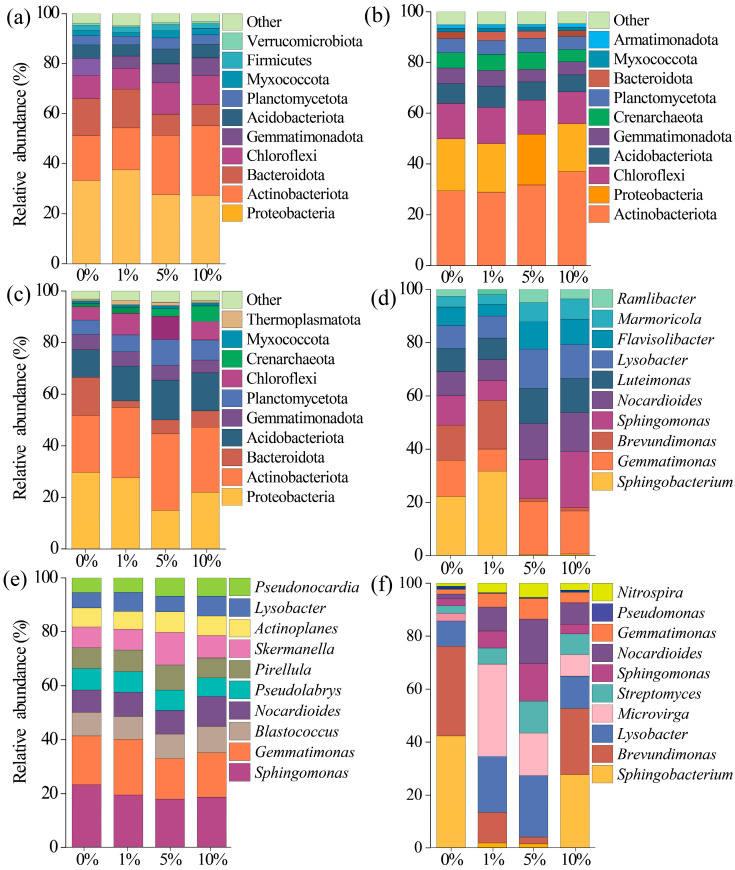
Dominant phyla and genus composition in soil bacterial communities (**a**–**f**). Panels correspond to soils S1 (**a**,**d**), S2 (**b**,**e**), and S3 (**c**,**f**).

The relative abundances of dominant bacterial phyla (Figure 3a–c) and classes (Figure 3d–f) in the three soils were significantly altered under MP treatments. At the phylum level, MPs significantly increased the relative abundance of Actinobacteriota and Chloroflexi, whereas Bacteroidota and Proteobacteria decreased in all three soils (*p* < 0.05). At the genus level, MPs increased the abundance of *Gemmatimonas*, *Sphingomonas*, *Nocardioides*, *Flavisolibacter*, and *Marmoricola* in S1. In S2, the abundance of *Nocardioides*, *Pseudonocardia*, and *Skermanella* increased, whereas in S3 MPs enhanced *Lysobacter* and *Microvirga*.

**Figure 4:**
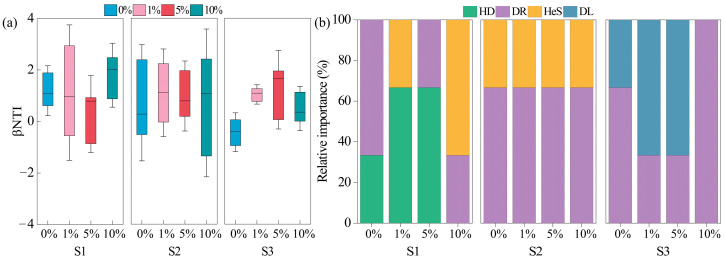
βNTI values of bacterial communities in three soils (**a**); changes in stochastic and deterministic processes of three soils (**b**).

The βNTI values under 0% MPs treatment in all soils were mainly between −2 and 2, indicating that stochastic processes dominated the bacterial community assembly (Figure 4a). After MP addition, several βNTI values in S1 and S2 exceeded 2, reflecting stronger deterministic processes. In S1, the deterministic assembly increased from 0% to 22% and reached 66% under 10% MPs. In contrast, deterministic processes in S2 were unaffected by MPs. In S3, the βNTI values under all MP treatments remained between −2 and 2 (Figure 4a). Specifically, in S1, the deterministic process of heterogeneous selection (HeS) prevailed after 10% MPs (Figure 4b). In S3, dispersal limitation (DL) dominated under 1% and 5% MPs, whereas drift (DR) dominated in the other treatments (Figure 4b).

**Figure 5:**
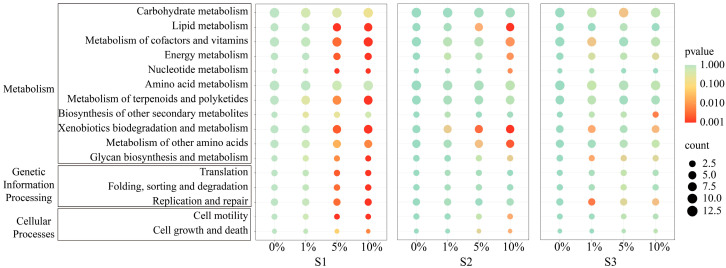
Changes in relative abundance of functional genes in soil bacteria.

In this study, PICRUSt2 software was used to predict the effects of MP pollution on soil bacterial functions, based on the Kyoto Encyclopedia of Genes and Genomes (KEGG) database and combined with bacterial 16S rRNA gene sequencing data. The comparison of secondary metabolic pathways across treatments suggested that 1% MPs exerted no significant effect on predicted functional pathway abundance in any soil type. In contrast, 5% and 10% MPs induced significant changes in S1 and S2, respectively (Figure 5). Specifically, in S1, 5% and 10% MPs reduced the relative abundance of pathways related to energy metabolism, nucleotide metabolism, replication and repair, cell motility, metabolism of cofactors and vitamins, metabolism of terpenoids and polyketides, and glycan biosynthesis. Conversely, lipid metabolism, xenobiotic degradation, protein folding, sorting and degradation, and translation pathways were enhanced (Figure 5). In S2, the predicted abundance of lipid metabolism, xenobiotic degradation, and amino acid metabolism pathways increased with 10% MPs (Figure 3c). In contrast, MP treatments had no significant effect on putative bacterial metabolic pathways in S3 (Figure 5).

### 3.5. Connections Between Environmental Variables and Soil Bacterial Communities

**Figure 6:**
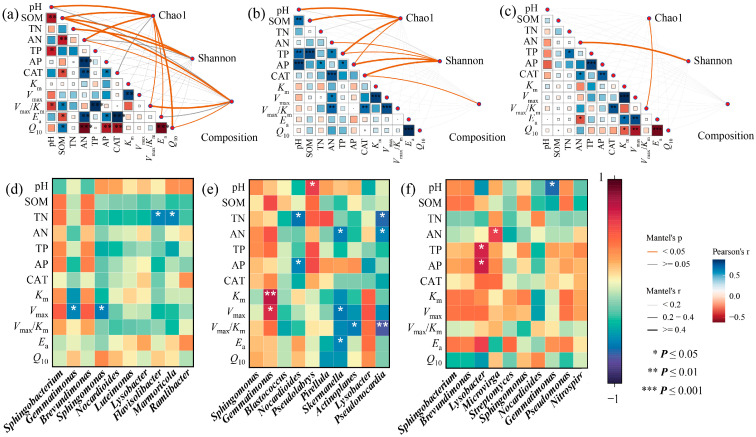
Mantel test results for correlations between bacterial communities and environmental variables in soils S1 (**a**), S2 (**b**), and S3 (**c**). Correlation between bacterial taxonomic levels and environmental variables in soils S1 (**d**), S2 (**e**), and S3 (**f**).

MP addition caused significant correlations among bacterial diversity, community structure, and soil physicochemical and enzymatic properties. In S1, SOM and pH were the two major drivers of bacterial diversity, whereas SOM and *V*_max_/*K*_m_ primarily shaped community structure (*p* < 0.05, Figure 6a). In S2, CAT was the dominant factor influencing both bacterial diversity and structure (*p* < 0.05, Figure 6b). In S3, AN and *V*_max_/*K*_m_ emerged as key drivers of bacterial diversity (*p* < 0.05, Figure 6c). Correlation analysis further revealed specific relationships between bacterial genera and soil environmental variables (Figure 6d–f). In S1, *Flavisolibacter* and *Marmoricola* were positively correlated with TN (*p* < 0.05, Figure 6d). In S2, Pseudolabrys was negatively correlated with pH, whereas *Pseudonocardia*, *Nocardioides*, and *Skermanella* were positively correlated with N content (*p* < 0.05, Figure 6e). In S3, Gemmatimonas exhibited a positive correlation with soil pH, and Lysobacter was negatively correlated with *p* content (*p* < 0.05, Figure 6f).

Enzyme activity was associated with kinetic and thermodynamic parameters. In S1, CAT activity was significantly correlated with *E*_a_ and *Q*_10_ (*p* < 0.05, Figure 4a). In S2 and S3, CAT activity was significantly correlated with *V*_max_/*K*_m_ (*p* < 0.05, Figure 6b,c).

## 4. Discussion

### 4.1. Response of CAT Activity and Enzymatic Reaction Characteristics in Three Soils to MPs

In this study, the CAT activity of the three soils with different pH values exhibited a consistent trend after MP exposure, which was characterized by stimulation at low concentrations and inhibition at high concentrations (Figure 1). This pattern reflects the typical “hormesis” effect of MPs and is aligned with previous reports. For example, in northern China, 7% and 14% MPs enhanced CAT activity, whereas 28% MPs suppressed CAT activity in farmland soils [16,49]. These results suggested that the response of enzyme activity to MPs followed a general rule irrespective of soil type, while the three soils differed in pH, and all belonged to the northeastern black soil region.

Ma et al. reported that *V*_max_ and *V*_max_/*K*_m_ could be utilized to evaluate the influence of antimony (Sb) on soil arylsulfatase activity and that soil characteristics such as pH strongly affect the toxicity of Sb to *V*_max_/*K*_m_ [50]. Building upon this evidence, the present study assessed the kinetic and thermodynamic parameters of CAT to determine whether MPs could exert distinct influences on enzymatic reactions in different soils, which served as an aspect rarely considered in MP-related research. The results revealed the evident variations in the kinetic and thermodynamic responses of the three soils after MP exposure. Notably, *E*_a_ and *Q*_10_ in acidic soil, *V*_max_/*K*_m_ in neutral soil and alkaline soil were significantly correlated with CAT activity (Figure 6a–c). The *V*_max_/*K*_m_ ratio represents the catalytic efficiency of the enzymatic process [51,52], and *E*_a_ represents the reaction activation energy [53,54]. These results indicated that although MPs consistently altered CAT activity in the three soils, the mechanisms regulating enzyme responses were not uniform. MPs may influence activity either by modifying the energy state of the enzymatic reaction or by altering the catalytic efficiency of the enzyme. The calculation of thermodynamic parameters *E*_a_ was derived from enzymatic kinetic data. These parameters primarily serve to describe trends in energy changes rather than to directly confirm specific molecular mechanisms. Consequently, their ecological significance requires further validation through more direct experimental approaches, such as molecular docking or proteomics.

Given the ongoing rise in global temperature, investigating the influence of temperature on soil enzyme activity and enzymatic reaction kinetics is of critical importance [55,56]. The interaction between MP pollution and climate change represents a major environmental challenge, with widespread consequences for ecosystems and human health. Increasing global temperatures intensify drought events, which significantly affects the abundance, transport, and distribution of MPs in soil systems [57,58]. The persistence of MPs in dry soils may disrupt biogeochemical cycling and impair essential ecosystem functions such as litter decomposition, soil aggregation, and nutrient cycling [59]. Therefore, the combined effects of MPs and climate feedback should be incorporated into environmental risk assessments [60,61]. The present results further demonstrated that MP addition significantly decreased *Q*_10_-*V*_max_/*K*_m_ in all three soils. *Q*_10_-*K*_m_ and *Q*_10_-*V*_max_ in acidic soils decreased under 5% and 10% MPs, whereas those in neutral and alkaline soils (except alkaline soil with 10% MPs) increased (Table 3). These findings indicate that MPs enhance the sensitivity of enzyme kinetic processes to temperature fluctuations, making them more responsive to thermal changes than the enzyme activity itself.

In future research, metagenomic technologies can be applied to analyze variations in the abundance of functional genes encoding enzymes. Synchrotron radiation technology can facilitate visualization of the spatial distribution of particles, soil colloids, and enzymes, thereby elucidating the intrinsic mechanisms through which particles induce alterations in enzyme kinetics. Moreover, changes in enzyme kinetic parameters (e.g., *Q*_10_-*V*_max_/*K*_m_) can be correlated with ecosystem functions, such as litter decomposition rate and nutrient cycling efficiency, to further substantiate the ecological relevance of the experimental findings.

### 4.2. Response of Bacteria in Three Soils to MPs

With an increase in MPs, notable shifts in the relative abundances of Actinobacteria, Proteobacteria, Chloroflexi, and Bacteroidetes were observed across all three soils (Figure 3a–c). Furthermore, the relative abundances of several bacterial genera exhibited significant variation following MP addition (Figure 3d–f). These taxa are generally associated with soil fertility, nutrient turnover, and decomposition of soil organic matter [62,63]. Such changes may be attributed to microbial feedback responses to the altered availability of carbon, nitrogen, and phosphorus under MP influence. In the present study, the relative abundances of *Flavisolibacter* and *Marmoricola* were positively correlated with N content in S1; *Pseudonocardia*, *Nocardioides*, and *Skermanella* in S2 exhibited similar *correlations*; and *Lysobacter* showed a significant correlation with *p* content in S3 (Figure 6d–f). These results were consistent with previous findings, indicating that MP-induced changes in soil physicochemical properties can strongly affect bacterial composition.

The impact of MPs on soil microbial communities is complex and dependent not only on the type, concentration, and particle size of MPs but also on soil properties [64]. Contrary to expectations, bacterial diversity responses differed among soils after MP addition. MPs increased bacterial diversity in acidic soils but suppressed diversity in neutral soils (Table 4), whereas the bacterial diversity of alkaline soil remained unchanged. These variations could be linked to microplastic-induced alterations in soil physicochemical processes. Specifically, MPs may accelerate the turnover of dissolved organic carbon and nutrients in acidic soil, thereby facilitating microbial colonization, enhancing *α*-diversity, and altering microbial community structure [65]. This mechanism explains the increase in *α*-diversity observed in acidic soil. In contrast, the decline in diversity in neutral soil may be attributed to the adverse effects of MPs on nutrient availability, including reductions in nitrogen, phosphorus, and total nutrients (Table 1). Our study demonstrated that MPs significantly altered the bacterial community structure only in acidic soil (Figure 2), suggesting that bacteria in acidic soils are more sensitive to environmental filtering imposed by MPs [66]. Under acidic conditions, MPs are more likely to adsorb metal ions and organic pollutants, thereby acting as “carriers of pollutants” and enhancing microbial toxicity [67]. From another perspective, MPs exert selective pressure on microbial communities, compelling shifts in their original assembly processes [68,69,70]. This selective pressure was particularly pronounced in acidic soils, driving microbial communities to shift toward deterministic processes (Figure 4a), with heterogeneous selection (HeS) dominating bacterial assembly (Figure 4b). As a result, bacterial communities in acidic soil exhibited stronger compositional divergence compared with other soils.

Changes in microbial communities can also influence metabolic functional diversity [71]. In this study, 16S rRNA gene data obtained on day 60 were annotated using the KEGG database for functional prediction. The abundances of various metabolism-related genes remained unchanged under 1% MP addition, but altered under 5% and 10% MP treatments. In acidic and neutral soils, multiple metabolic pathways were significantly affected, whereas the metabolic profile of alkaline soil remained largely undisturbed (Figure 5). The prediction of bacterial functions facilitates in-depth verification of changes in soil bacterial communities, with the magnitude and direction of such changes varying across soil types. Notably, in acidic and neutral soils, MP exposure significantly increased the abundances of genes related to lipid metabolism and xenobiotic biodegradation. Xenobiotic degradation pathways have been associated with contaminant degradation [72,73], suggesting that bacteria enriched under MP conditions can utilize plastic polymers or their additives as carbon sources. Lipid metabolism pathways, particularly those related to fatty acid, wax, and cutin degradation, were also stimulated, which may alter hydrophobic compound turnover on soil particle surfaces [74].

In this study, the physicochemical properties and enzyme activities of acidic and neutral soils exhibited strong correlations with bacterial communities, whereas in alkaline soil, only AN and *V*_max_/*K*_m_ were significantly associated with bacterial diversity (Figure 6a–c). These findings indicated that the bacterial community composition in acidic soil presented the most pronounced response to MPs, followed by neutral soil, while the weakest response occurred in alkaline soil.

In acidic soil, the bacterial diversity index correlated with *V*_max_/*K*_m_, *E*_a_, and *Q*_10_. In neutral soil, the bacterial diversity index was associated with CAT. In alkaline soil, the bacterial diversity index was correlated primarily with *V*_max_/*K*_m_. Moreover, CAT activity across the three soils also exhibited significant associations with these parameters (Figure 6a–c). The Mantel test results further indicated that SOM and *V*_max_/*K*_m_ were the main determinants of bacterial community composition in acidic soil (Figure 6a). This observation could be reasonable because MPs increased the soil carbon content, thereby enhancing carbon cycling and driving shifts in microbial distribution and composition [75]. Additionally, our results demonstrated that the abundance of *Gemmatimonas*, *Sphingomonas*, and *Nocardioides*, which are involved in the carbon cycle in acidic soils, increased following MP introduction (Figure 6d). In neutral soil, CAT activity is a key factor influencing the bacterial community composition (Figure 6b). Among the major indicators affecting bacteria, the thermodynamic parameters of enzyme were also significant, suggesting that MPs exerted consistent effects on enzymatic activity across soils, whereas their impacts on microorganisms varied depending on the soil type. Such variability appears to be associated with changes in the intrinsic enzymatic parameters. Soil pH is a crucial determinant of soil bacterial community structure [76]. The variations in soil pH likely explained the observed inconsistencies in the driving forces across the three soils. In addition, differences in crop types may also contribute to discrepancies between soils [77]. It should be noted that due to practical constraints, this study did not conduct comprehensive polymer characterization of the LDPE used, nor did it establish a control group to distinguish the effects of the polymer matrix from leachates, making it difficult to clearly separate the independent impacts of the LDPE polymer matrix itself and surface leachates on soil microorganisms; this limits the in-depth analysis of the ecological mechanism of microplastics to a certain extent, and future studies could add this control group to accurately elucidate the mechanism of microplastics’ effects.

## 5. Conclusions

CAT activity in all three soils exhibited a consistent response pattern following MP addition, which was characterized by an initial increase followed by a decline. MPs imposed differential effects on the physicochemical properties and kinetic/thermodynamic parameters of the soils, with the magnitude of influence depending on soil type and MP concentration. MP treatments markedly altered bacterial diversity and functional gene abundance in acidic and neutral soils, whereas no significant shifts were observed in alkaline soil. Furthermore, MPs modified the distribution of bacterial phyla and genera across the three soils. Under conditions dominated by stochastic processes, MPs enhanced deterministic processes exclusively in acidic soil. Upon MP entry into soils, CAT activity and bacterial indices were correlated with kinetic and thermodynamic parameters across the three soil types. These findings suggested that the differential responses of soils to MPs were more strongly reflected in microbial community dynamics than in enzyme activity alone. Therefore, to evaluate the ecological effects of MPs on soils, both microbial responses and the kinetic and thermodynamic characteristics of enzymes should be considered. The MP concentrations used in this study are significantly higher than the actual levels of MPs in naturally occurring soils (< 0.1%), potentially leading to discrepancies between the study results and the actual effect intensity of MPs in natural soil environments. Meanwhile, the 60-day incubation period only captures microbial responses to short-term MP exposure, making it difficult to track adaptive changes in microbial communities during long-term pollution. Thus, future studies may incorporate MP treatments at environmentally realistic concentrations to enhance the ecological relevance of findings, and extend the incubation period to elucidate dynamic microbial responses under long-term MP pollution.

## Figures and Tables

**Table 1:** Physicochemical properties in three soils with MP addition.

Soil Type	Treatment	pH	SOM (g kg^−1^)	TN (g kg^−1^)	AN (mg kg^−1^)	TP (g kg^−1^)	AP (mg kg^−1^)
S1	0%	5.41 ± 0.05 c	26.25 ± 0.99 a	1.24 ± 0.02 a	56.70 ± 0.70 d	1.845 ± 0.02 a	137.898 ± 0.45 d
	1%	5.61 ± 0.10 b	23.39 ± 0.00 b	1.23 ± 0.04 a	68.13 ± 1.07 a	1.829 ± 0.01 b	163.469 ± 0.68 a
	5%	5.80 ± 0.01 a	22.24 ± 0.99 b	1.22 ± 0.02 a	66.03 ± 1.07 b	1.819 ± 0.01 b	148.688 ± 0.45 b
	10%	5.87 ± 0.02 a	22.24 ± 0.99 b	1.20 ± 0.02 a	62.77 ± 1.07 c	1.718 ± 0.01 c	143.012 ± 0.35 c
S2	0%	6.64 ± 0.04 a	12.50 ± 0.99 a	1.10 ± 0.03 a	34.77 ± 0.81 a	1.115 ± 0.01 a	104.909 ± 0.35 a
	1%	6.50 ± 0.01 c	10.20 ± 0.99 b	1.09 ± 0.01 a	35.93 ± 0.40 a	1.050 ± 0.01 b	103.054 ± 0.52 b
	5%	6.59 ± 0.01 b	9.40 ± 0.40 b	1.09 ± 0.01 a	32.20 ± 0.70 b	1.001 ± 0.01 c	103.897 ± 0.35 b
	10%	6.53 ± 0.01 c	9.86 ± 0.40 b	1.07 ± 0.02 a	32.67 ± 0.81 b	1.003 ± 0.01 c	101.368 ± 0.54 c
S3	0%	7.49 ± 0.01 a	23.96 ± 0.99 a	1.18 ± 0.02 c	66.50 ± 0.70 b	0.862 ± 0.04 a	42.159 ± 0.74 a
	1%	7.46 ± 0.01 a	23.73 ± 2.26 a	1.25 ± 0.02 b	71.17 ± 1.07 a	0.783 ± 0.01 b	43.328 ± 0.21 a
	5%	7.52 ± 0.01 a	23.39 ± 1.72 a	1.24 ± 0.01 ab	67.20 ± 0.70 b	0.750 ± 0.02 c	25.883 ± 0.27 b
	10%	7.56 ± 0.02 a	22.81 ± 0.99 a	1.21 ± 0.01 a	66.03 ± 1.07 b	0.685 ± 0.01 d	23.815 ± 0.21 c

Data are presented as mean ± standard deviation of three independent replicate experiments. Lowercase letters indicate significant differences among MP treatment groups within the same soil (*p* < 0.05).

**Table 2:** Two-factor variance analysis of the soil type, and MP concentration on enzyme parameters and soil microflora (F-value).

Index	Soil Type (S)	Concentration (C)	S × C
CAT	34,300.695 **	165.831 **	120.605 **
*K*_m_	11.195 **	3.437 *	4.903 *
*V*_max_	870.534 **	9.374 **	6.482 **
*V*_max_/*K*_m_	3782.430 **	11.590 **	16.727 **
*E*_a_	1982.409 **	32.682 **	212.702 **
Δ*G*	28,329.040 **	105.907 **	55.812 **
Chao 1	53.634 **	12.386 **	17.119 **
Shannon	14.313 **	1.290	2.200

An asterisk (*) indicated statistically significant at *p* < 0.05 and a double asterisk (**) indicated statistically significant at *p* < 0.01.

**Table 3:** Temperature sensitivity of CAT and kinetic parameters in three soils with MP addition.

Soil Type	Index	0%	1%	5%	10%
S1	*Q*_10_-CAT	0.49 ± 0.02 a	0.45 ± 0.03 a	0.48 ± 0.00 a	0.48 ± 0.01 a
	*Q*_10_-*K*_m_	0.27 ± 0.03 a	0.24 ± 0.01 a	0.15 ± 0.01 b	0.14 ± 0.03 b
	*Q*_10_-*V*_max_	7.88 ± 0.86 a	7.22 ± 0.23 a	4.71 ± 0.23 b	2.90 ± 0.96 c
	*Q*_10_-*V*_max_/*K*_m_	10.71 ± 0.24 a	7.59 ± 0.32 c	6.99 ± 0.36 c	8.54 ± 0.55 b
S2	*Q*_10_-CAT	0.62 ± 0.02 a	0.63 ± 0.01 a	0.63 ± 0.00 a	0.61 ± 0.10 a
	*Q*_10_-*K*_m_	0.23 ± 0.02 b	0.28 ± 0.04 b	0.46 ± 0.04 a	0.49 ± 0.07 a
	*Q*_10_-*V*_max_	2.94 ± 0.42 b	3.38 ± 0.71 b	5.66 ± 0.09 a	6.43 ± 0.64 a
	*Q*_10_-*V*_max_/*K*_m_	5.51 ± 0.48 a	4.78 ± 0.28 a	4.41 ± 0.26 a	4.45 ± 0.68 a
S3	*Q*_10_-CAT	0.62 ± 0.02 ab	0.59 ± 0.04 b	0.64 ± 0.01 a	0.65 ± 0.01 a
	*Q*_10_-*K*_m_	0.13 ± 0.02 b	0.14 ± 0.06 b	0.27 ± 0.06 a	0.09 ± 0.01 b
	*Q*_10_-*V*_max_	7.32 ± 1.36 b	6.73 ± 1.13 b	11.16 ± 1.73 a	7.28 ± 0.58 b
	*Q*_10_-*V*_max_/*K*_m_	14.97 ± 0.44 a	13.59 ± 0.37 b	12.78 ± 0.40 b	8.64 ± 1.01 c

Data are presented as mean ± standard deviation of three independent replicate experiments. Data are the mean of temperature sensitivity at 15–25 °C, 25–35 °C, and 35–45 °C. Lowercase letters indicate significant differences among MP treatment groups within the same soil (*p* < 0.05).

**Table 4:** Alpha diversity indexes of bacteria in three soils with MP addition.

Soil Type	Treatments	Chao1	Shannon
S1	0%	1829.4 ± 202.56 c	8.935 ± 0.15 b
	1%	3014.2 ± 344.24 b	9.010 ± 0.09 a
	5%	3568.1 ± 10.86 ab	9.330 ± 0.03 a
	10%	3777.6 ± 17.50 a	9.260 ± 0.03 a
S2	0%	4089.4 ± 50.97 a	9.530 ± 0.03 a
	1%	3775.7 ± 18.83 b	9.500 ± 0.01 ab
	5%	3825.7 ± 21.56 b	9.403 ± 0.02 b
	10%	3762.8 ± 49.96 b	9.250 ± 0.04 b
S3	0%	3945.5 ± 184.31 ab	9.555 ± 0.00 a
	1%	4063.4 ± 121.02 a	9.353 ± 0.30 a
	5%	3634.0 ± 73.63 b	9.647 ± 0.02 a
	10%	4218.3 ± 40.65 a	9.600 ± 0.09 a

Data are presented as mean ± standard deviation of three independent replicate experiments. Lowercase letters indicate significant differences among MPs treatment groups within the same soil (*p* < 0.05).

## Data Availability

Raw data from the experiments are available for individual use upon reasonable request to the corresponding author (Z.Y.).

## Supplementary Materials

The following supporting information can be downloaded at: https://www.mdpi.com/article/10.3390/toxics13121005/s1, Figure S1: The Lineweaver–Burk graph representing different MPs treatments. The horizontal axis corresponds to the inverse of substrate concentration (1/S); the vertical axis corresponds to the inverse of the enzymatic reaction rate (1/V). R^2^ corresponds to the degree of fit of the linear equation.; Table S1: Effects of MPs on Catalase (CAT) Activity in Three Soil Types under Different Incubation Days.
