# Supplementary material for: Do Soil pH Levels Drive the Responses of Catalase Activity and Bacterial Communities to Microplastics? A Case Study in Mollisols
Source: Toxics. 2025 Nov 21;13(12):1005. doi: 10.3390/toxics13121005 (PMC12736934; doi:10.3390/toxics13121005)
Supplement: Supplementary file 1 [file toxics-13-01005-s001.zip › Supplementary Figure.pdf]

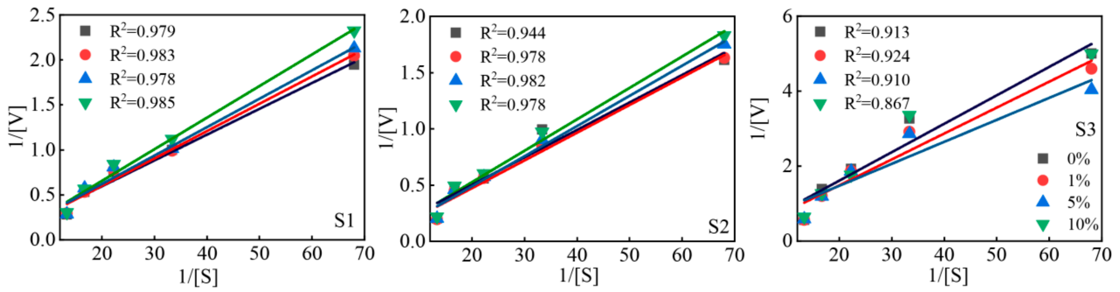

Figure S1. The Lineweaver–Burk graph representing different MPs treatments. The horizontal axis corresponds to the inverse of substrate concentration (1/S); the vertical axis corresponds to the inverse of the enzymatic reaction rate (1/V). R^2 corresponds to the degree of fit of the linear equation.
