# Supplementary material for: Do Soil pH Levels Drive the Responses of Catalase Activity and Bacterial Communities to Microplastics? A Case Study in Mollisols
Source: Toxics. 2025 Nov 21;13(12):1005. doi: 10.3390/toxics13121005 (PMC12736934; doi:10.3390/toxics13121005)
Supplement: Supplementary file 1 [file toxics-13-01005-s001.zip › Supplementary Table.pdf]

Table S1. Effects of MPs on Catalase (CAT) Activity in Three Soil Types under Different Incubation Days

Soil type	Day	CAT (mL g^{-1})			
		0%	1%	5%	10%
S1	0	1.900±0.00	1.793±0.03	1.667±0.06	1.727±0.02
	1	2.520±0.11	2.120±0.03	2.300±0.00	2.191±0.01
	3	1.938±0.04	1.970±0.08	1.850±0.06	1.870±0.00
	7	2.213±0.07	2.139±0.08	1.848±0.02	1.745±0.05
	15	2.398±0.02	2.231±0.04	2.053±0.03	1.955±0.01
	30	2.556±0.01	2.232±0.03	2.252±0.03	2.209±0.02
	60	3.012±0.01	2.700±0.01	2.469±0.00	2.280±0.01
	75	3.224±0.06	2.666±0.05	2.435±0.07	2.203±0.04
	90	3.137±0.03	2.670±0.09	2.328±0.12	2.274±0.03
S2	0	0.627±0.01	0.600±0.02	0.633±0.02	0.607±0.03
	1	0.589±0.00	0.572±0.00	0.568±0.00	0.609±0.05
	3	0.580±0.03	0.558±0.04	0.676±0.02	0.662±0.03
	7	0.539±0.00	0.547±0.03	0.583±0.03	0.550±0.01
	15	0.514±0.03	0.541±0.00	0.545±0.05	0.507±0.00
	30	0.533±0.00	0.541±0.03	0.521±0.01	0.473±0.09
	60	0.653±0.01	0.746±0.00	0.794±0.01	0.715±0.00
	75	0.583±0.04	0.583±0.09	0.562±0.04	0.542±0.01
	90	0.573±0.07	0.554±0.00	0.566±0.07	0.504±0.04
S3	0	2.753±0.08	2.780±0.03	2.700±0.05	2.693±0.03
	1	2.097±0.02	2.153±0.14	2.094±0.02	1.893±0.03
	3	2.820±0.05	2.686±0.02	2.622±0.04	2.527±0.00
	7	3.020±0.08	3.419±0.07	3.207±0.16	3.110±0.05
	15	3.492±0.04	3.420±0.02	3.173±0.00	3.212±0.00
	30	3.890±0.03	3.551±0.03	3.375±0.00	3.038±0.00
	60	3.793±0.00	3.871±0.01	3.687±0.03	3.340±0.02
	75	3.784±0.04	3.472±0.05	3.668±0.00	3.469±0.07
	90	3.557±0.07	3.564±0.08	3.044±0.09	3.284±0.03

Data are presented as mean ± standard deviation of three independent replicate experiments.
