# Supplementary figures and images for: Do Soil pH Levels Drive the Responses of Catalase Activity and Bacterial Communities to Microplastics? A Case Study in Mollisols
Source: Toxics. 2025 Nov 21;13(12):1005. doi: 10.3390/toxics13121005 (PMC12736934; doi:10.3390/toxics13121005)

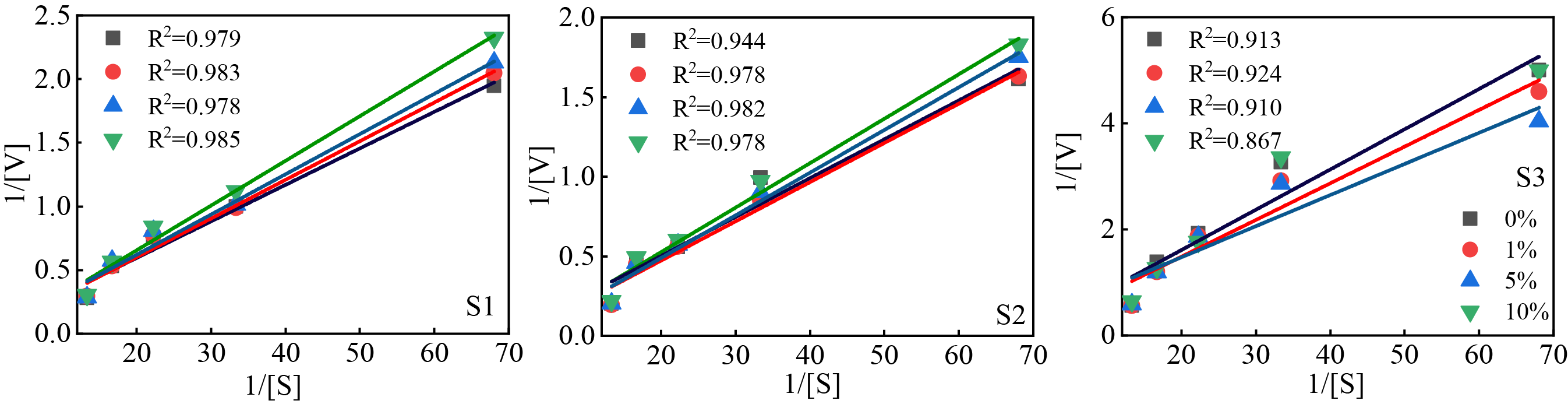

Supplement: Supplementary file 1 [file toxics-13-01005-s001.zip › Figure S1.png]
